# Corporate Capture of the laws application in Colombia: a new form of capture for public policy actions

**DOI:** 10.1093/eurpub/ckac129.090

**Published:** 2022-10-25

**Authors:** H Salcedo Fidalgo

**Affiliations:** Main, FIAN Colombia, Cartagena de Indias, Colombia

## Abstract

The law popularly known by the title ‘Junk Food’, was approved in Colombia in 2021 after years of advocacy from the Civil Society. Many authors as M. Mialon (2019, 2020, 2021) have documented the level of actions to intervene in the legislative space of the Parliament in Colombia created by the food industries to change the terms of the content of this law. Nowadays and after the approval of the text interpreted as a victory for Civil Society, Colombia is confronted with the re-regulation of the law, leading to a new form of capture: the capture of the spaces of decision-making actors in public health policy in the country. We present an analysis of the case from a follow-up study designed by our organization FIAN Colombia (human rights organization that advocates for the right to adequate food and nutrition). The data used in this process of monitoring and action research comes from the observational data of the advocacy scenario made during the debates of the law in the Colombian parliament, based on the proceedings of the work meetings with congressmen, academia and civil society. This research-action process makes it clear that the collective action of advocacy from civil society manages to mobilize conditions to denounce the corporate capture of decision-making spaces and the execution of public health policies, and generates pressures based on research free of conflict of interest to reduce this capture. Similarly, there is a capacity to demand accountability that was generated from this process in Colombia, and that turns out to be a pilot experience for other regions.

